# Versatile Strategy for Electrophoretic Deposition of Polyvinylidene Fluoride-Metal Oxide Nanocomposites

**DOI:** 10.3390/ma14247902

**Published:** 2021-12-20

**Authors:** Qinfu Zhao, Xinqian Liu, Stephen Veldhuis, Igor Zhitomirsky

**Affiliations:** 1 Department of Materials Science and Engineering, McMaster University, Hamilton, ON L8S 4L7, Canada; zhaoq36@mcmaster.ca (Q.Z.); liux234@mcmaster.ca (X.L.); 2 Department of Mechanical Engineering, McMaster University, Hamilton, ON L8S 4L7, Canada; veldhu@mcmaster.ca

**Keywords:** polyvinylidene fluoride, electrophoretic deposition, manganese dioxide, titanium dioxide, nickel, iron, superparamagnetism, nanocomposite

## Abstract

Polyvinylidene fluoride (PVDF) is an advanced functional polymer which exhibits excellent chemical and thermal stability, and good mechanical, piezoelectric and ferroelectic properties. This work opens a new strategy for the fabrication of nanocomposites, combining the functional properties of PVDF and advanced inorganic nanomaterials. Electrophoretic deposition (EPD) has been developed for the fabrication of films containing PVDF and nanoparticles of TiO_2_, MnO_2_ and NiFe_2_O_4_. An important finding was the feasibility of EPD of electrically neutral PVDF and inorganic nanoparticles using caffeic acid (CA) and catechol violet (CV) as co-dispersants. The experiments revealed strong adsorption of CA and CV on PVDF and inorganic nanoparticles, which involved different mechanisms and facilitated particle dispersion, charging and deposition. The analysis of the deposition yield data, chemical structure of the dispersants and the microstructure and composition of the films provided an insight into the adsorption and dispersion mechanisms and the influence of deposition conditions on the deposition rate, film microstructure and composition. PVDF films provided the corrosion protection of stainless steel. Overcoming the limitations of other techniques, this investigation demonstrates a conceptually new approach for the fabrication of PVDF-NiFe_2_O_4_ films, which showed superparamagnetic properties. The approach developed in this investigation offers versatile strategies for the EPD of advanced organic-inorganic nanocomposites.

## 1. Introduction

Poly (vinylidene fluoride) (PVDF) is an advanced functional polymer, which has outstanding chemical resistance, thermal stability, mechanical strength, piezoelectric and ferroelectric properties [1,2,3,4,5,6]. Significant interest has been generated in the fabrication of advanced nanocomposites, combining functional properties of PVDF and inorganic nanomaterials [7]. Flexible PVDF–MnO_2_ nanocomposites showed enhanced piezoelectric and mechanical properties [8]. Moreover, such nanocomposites are of particular interest for energy storage applications [9] due to the outstanding pseudocapacitive properties of MnO_2_. PVDF–TiO_2_ nanocomposites have been developed [10] for application in separation membranes, which showed enhanced fouling resistance. Ferrimagnetic NiFe_2_O_4_ nanoparticles were added to PVDF [11] in order to obtain composites, combining the ferroelectric and piezoelectric properties of PVDF and the magnetic properties of NiFe_2_O_4_. The addition of NiFe_2_O_4_ nanoparticles resulted in enhanced formation of ferroelectric β phase of PVDF [12]. Nanocomposites of PVDF and NiFe_2_O_4_ showed magnetoelectric properties, which were analyzed by investigating the influence of the magnetic field on ferroelectric properties [13]. Many applications of PVDF–metal oxide nanocomposites involved the use of films and significant attention has been focused on the development of film deposition techniques. The combination of organic and inorganic materials in composites offers a possibility of fabricating multifunctional materials with advanced properties [14,15,16].

Colloidal film deposition techniques are of particular interest for nanotechnology of composites due to the possibility of composite design on the nanometric scale. Currently, there are several methods which are commonly used for the fabrication of PVDF films: solvent casting, spin coating, and electrospinning [17]. Such techniques are based on the use of PVDF solutions. PVDF dissolves in a few non-ecofriendly organic solvents such as dimethylformamide, dimethyl sulfoxide, N-methyl pyrrolidone, or dimethylacetamide, and there is a significant interest in replacing such solvents with more environmentally friendly solvents.

Electrochemical deposition methods are very promising for the deposition of PVDF and composite films. Various electrochemical strategies have been utilized for the fabrication of polymer–metal oxide nanocomposites [18,19]. Electrophoretic deposition (EPD) is a colloidal technique, which offers advantages for the deposition of nanocomposites due to high deposition rate, simple control of film thickness, and uniformity [20,21,22,23,24,25,26]. This technique is especially important for the deposition of nanoparticles as uniform monolayers or multilayers on substrates of complex shape [27]. Several previous investigations reported the EPD of pure PVDF films in different organic solvents without the use of surfactants [28,29,30]. It should be noted that PVDF is an electrically neutral polymer, and the use of charged surfactants facilitates the control of particle charge and EPD yield. Co-surfactants are highly desired for the co-deposition of PVDF with inorganic nanoparticles. Inorganic nanoparticles are prone to forming agglomerates due to their high surface energy. It is challenging to disperse magnetic nanoparticles due to their magnetic interactions, which promote agglomeration. In a previous investigation [31], bile salts were used as charged surfactants for PVDF. However, bile salts show poor adsorption on metal oxide surfaces and their applications for EPD of PVDF–metal oxide films present difficulties. The EPD of metal oxide nanoparticles requires the use of efficient surfactants with a very small size and strong adsorption on the particle surface. It is challenging to develop co-dispersants with strong adsorption on metal oxide nanoparticles and hydrophobic PVDF particles. In our investigation we addressed this problem using a biomimetic strategy, which is based on the use of catecholate-type molecules as dispersants.

The goal of this investigation was the fabrication of PVDF–metal oxide films by EPD using chelating catecholate molecules as co-dispersants for PVDF and metal oxide nanoparticles. Due to significant interest in the fabrication of PVDF composites containing MnO_2_, TiO_2_ and NiFe_2_O_4_ nanoparticles, we selected such nanomaterials as model metal oxides of different types for the feasibility studies, which showed the versatility of our approach. The biomimetic approach of our investigation was based on the analysis of the mechanism of mussel protein adhesion to different surfaces, which involves the super-strong catecholate type bonding to inorganic surfaces. The investigations of deposition efficiency provided an insight into the influence of the chemical structure of the dispersant molecules and charged groups on their adsorption on PVDF and metal oxide nanoparticles and related adsorption mechanisms. We demonstrate the feasibility of deposition of nanocomposite films using ethanol as a non-toxic solvent and report valuable properties of the deposited films, such as corrosion protection and superparamagnetic properties. Following the work objective we investigated the microstructure, composition, and properties of the films deposited at different conditions. 

## 2. Materials and Methods

### 2.1. Materials

Poly (vinylidene fluoride) (PVDF) and catechol violet (CV) were purchased from Alfa Aesar (Haverhill, MA, USA). Caffeic acid (CA), titanium oxide (TiO_2_, 21 nm) nanopowder, iron nickel oxide (NiFe_2_O_4_, < 50 nm) nanopowder, and KMnO_4_ were purchased from Aldrich (St. Louis, MO, USA). Nanoparticles of MnO_2_ with an average size of 50 nm were prepared by a chemical reduction of KMnO_4_ aqueous solutions with ethanol, as was described in the previous investigation [32]. The nanoparticles prepared by this method were amorphous [32]. 

### 2.2. Film Deposition

PVDF particles were dispersed in pure ethanol using CA or CV dispersants. The concentration of PVDF was 5–10 g L^−1^. The concentration of CA or CV was 0.2–1 g L^−1^. TiO_2_, MnO_2,_ and NiFe_2_O_4_ were added for the fabrication of composite films, and their concentrations were 1–3 g L^−1^. EPD was performed using these suspensions after ultrasonicating and stirring. A stainless-steel substrate (304 type, 20 × 30 × 0.1 mm^3^) and a platinum sheet (20 × 30 × 0.1 mm^3^) served as a working electrode and counter electrode, respectively, with 15 mm separation in electrochemical deposition cell for EPD. A deposition voltage of 20–100 V was applied for 5 min. EPD resulted in the accumulation of particles at the substrate surface. The films were dried at room temperature and then annealed at 200 °C for 1 h. The annealing process was essential for the formation of dense films.

### 2.3. Characterization

Zeta-potential measurements were performed using the mass transport method [33]. Electrochemical studies were performed in 3% aqueous NaCl solution using a potentiostat (PARSTAT 2273, Princeton Applied Research, Oak Ridge, TN, USA) and a 3-electrode cell with coated or uncoated stainless steel as a working electrode, a platinum counter electrode, and a saturated calomel electrode (SCE) as a reference electrode. Potentiodynamic studies were conducted at a sweeping rate of 1 mV s^−1^ from the negative to positive direction and obtained data was presented in Tafel plots. Electrochemical impedance spectroscopy (EIS) studies were preformed under a sinusoidal excitation voltage of 10 mV in the frequency range of 10 mHz–10 kHz. A JEOL JSM-7000 F microscope (Tokyo, Japan) was used for scanning electron microscopy (SEM) investigations. Talos 200X microscope (Waltham, MA, USA) was used for transmission electron microscopy (TEM) studies. FTIR studies were performed using a Bruker Vertex 70 spectrometer (Billerica, MA, USA). X-ray diffraction (XRD) was carried out using a Nicolet I2 powder diffractometer with monochromatized Co Kα radiation. Magnetic measurements were conducted using a Quantum Design PPMS-9 system (San Diego, CA, USA).

## 3. Results

**Figure 1:**
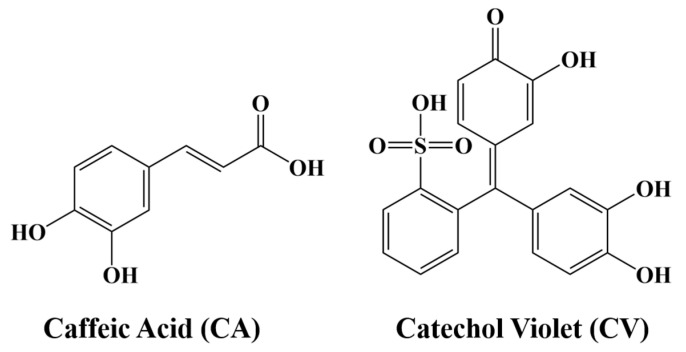
Chemical structures of dispersants.

TEM investigations showed that as-received PVDF particles were of spherical shape with a diameter of about 200 nm (Figure S1A,B). A significant challenge in the EPD of PVDF is obtaining stable suspensions of charged PVDF particles. This can be achieved using efficient dispersants, which must be adsorbed on the particle surface to impart colloidal stability and electric charge. Difficulties are related to poor adsorption of traditional dispersants on the surface of chemically inert electrically neutral hydrophobic PVDF particles. Another challenge is to find co-dispersants suitable for the co-dispersion of PVDF with metal oxide nanoparticles. Previous investigations showed that mutual repulsion of charged particles or polymer macromolecules at the electrode surface can prevent their deposition [19]. Particle coagulation and deposit formation at the electrode surface are influenced by the properties of the dispersant and local pH changes at the electrode [19]. Such challenges were addressed using CV and CA as dispersants (Figure 1).

The chemical structures of CV and CA contain a catechol group with two phenolic OH groups bonded to adjacent carbon atoms of the aromatic ring. The catecholate molecules were chosen due to the ability of catechol bonding to metal atoms on material surfaces [34]. The interest in dispersant with catechol anchoring groups resulted from the investigation of mechanism of mussel protein adsorption on rock surfaces in see water [34]. The catecholate adsorption involves two phenolic OH groups. Various bonding mechanisms are based on bidentate bridging or chelating bonding in the inner sphere or outer sphere modes [34].

The catechol adsorption on different surfaces is influenced by solvents, the nature of the surface atoms, and the chemical structure of the material. CA is a monoaromatic molecule, whereas the chemical structure of CV contains three aromatic groups. The anionic properties of CA and CV are related to their COOH and SO_3_H groups, respectively.

**Figure 2:**
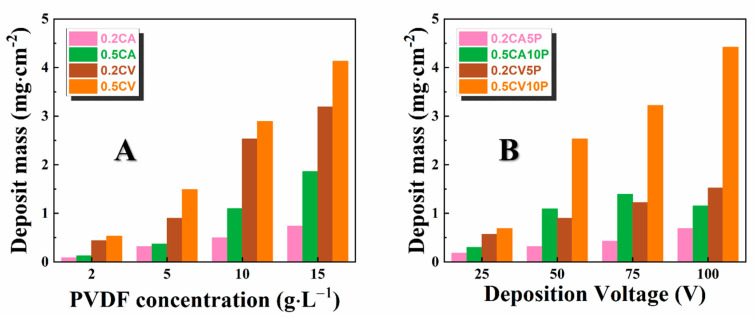
(**A**) Deposit mass versus PVDF concentration for suspensions, containing 0.2 g L^−1^ CA (0.2 CA), 0.5 g L^−1^ CA (0.5 CA), 0.2 g L^−1^ CV (0.2 CV), 0.5 g L^−1^ CV (0.5 CV) at a deposition voltage of 50 V and a deposition time of 5 min and (**B**) deposit mass versus deposition voltage for suspensions containing 0.2 g L^−1^ CA and 5 g L^−1^ PVDF (0.2 CA5P); 0.5 g L^−1^ CA and 10 g L^−1^ PVDF (0.5 CA10P); 0.2 g L^−1^ CV and 5 g L^−1^ PVDF (0.2 CV5P); and 0.5 g L^−1^ CV and 10 g L^−1^ PVDF (0.5 CV10P) for deposition time of 5 min.

In this investigation, CA and CV were used as dispersing and charging agents for EPD of PVDF powder from its suspension in ethanol. PVDF suspensions in ethanol were unstable and quick sedimentation was unavoidable even with ultrasonic treatment or stirring. No deposition was achieved from pure PVDF suspension because PVDF powder is electrically neutral. The addition of CA and CV molecules resulted in improved stability of PVDF suspensions by electrostatic stabilization, and anodic films were deposited by EPD. It was hypothesized that CA or CV molecules dissociated in ethanol, adsorbed on PVDF particles, and their deprotonated anionic groups imparted a negative charge to the PVDF particles. As a result, the negatively charged PVDF particles were deposited anodically from the suspensions containing catecholate molecules as additives. The deposition yield measurements indicated that the film mass increased significantly with increasing concentration of PVDF powder (Figure 2A). The PVDF particles in 10 g L^−1^ PVDF suspensions, containing 0.5 g L^−1^ CA and 0.5 L^−1^ CV showed zeta potentials of −11 mV and −26 mV, respectively.

CA and CV catecholate molecules showed sufficient charging and dispersing effects in a wide range of PVDF concentrations. The PVDF films produced with CV had higher deposit mass than that with CA, which indicated that CV allowed higher deposition efficiency than CA. It was suggested that adsorption of CA and CV molecules on PVDF particles involved hydrophobic interactions of the aromatic rings of the molecules with hydrophobic PVDF surfaces. The larger number of aromatic rings in the CV structure resulted in stronger interactions, which allowed for better adsorption of CV and enhanced PVDF dispersion and charging. Figure 2B shows deposit mass versus deposition voltage dependencies. The deposit mass presented increasing trends with increasing deposition voltage, indicating higher efficiency of particles’ motion and packing on the substrates. Therefore, the mass of the deposited PVDF films can be varied and controlled by EPD bath composition and deposition voltage.

**Figure 3:**
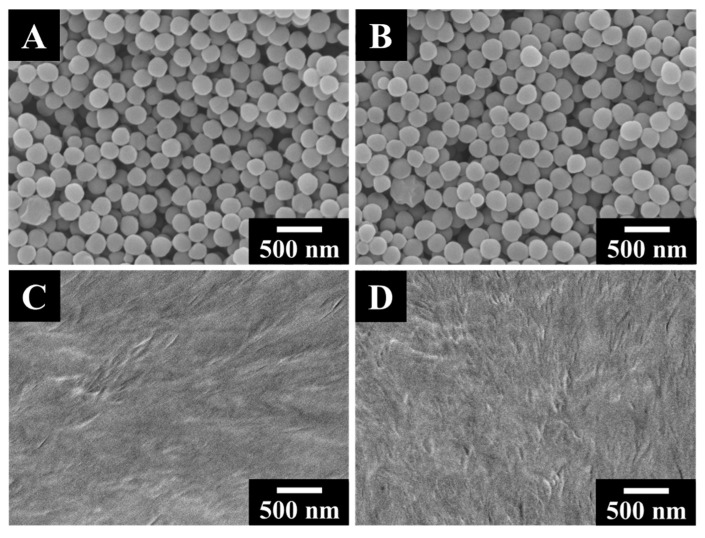
SEM images of films at different magnifications prepared from suspensions containing 10 g L^−1^ PVDF with (**A**,**C**) 0.5 g L^−1^ CA or (**B**,**D**) 0.5 g L^−1^ CV at a deposition voltage of 50 V for 5 min; (**A**,**B**) as-deposited and (**C**,**D**) annealed at 200 °C.

Morphologies of the as-deposited and heat-treated PVDF films fabricated using CA and CV were analyzed by SEM. Figure 3A,B shows that the as-deposited spherical PVDF powder was relatively densely packed on the stainless steel 304 substrates, offering good coverage of the substrates. However, there were still some nanosized voids between the PVDF spheres. After heat treatment at the temperature right above the melting point of PVDF for 1 h, PVDF spherical particles were completely melted and re-solidified into uniform and crack-free films (Figure 3C,D).

**Figure 4:**
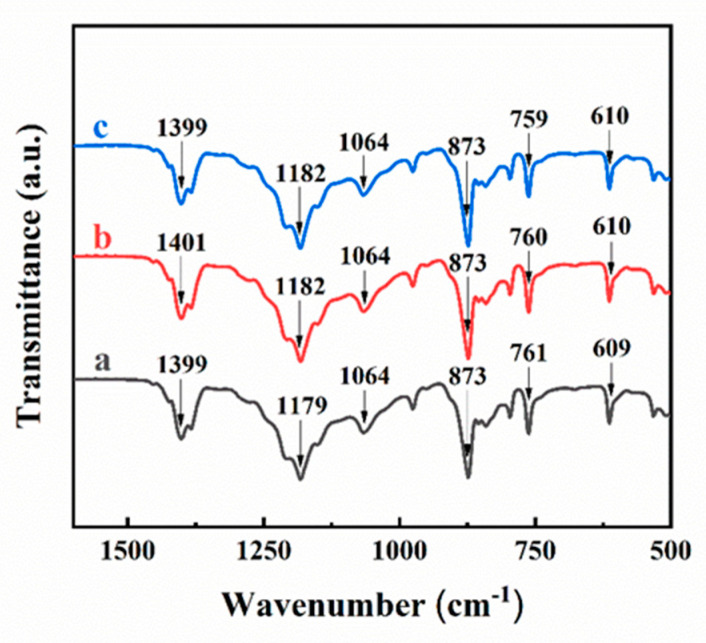
FTIR spectra of (a) as-received PVDF and (b,c) as-deposited PVDF, prepared from 0.5 g L^−1^ (b) CA or (c) CV solution containing 10 g L^−1^ PVDF at a deposition voltage of 50 V during 5 min.

The as-deposited PVDF films prepared using CA and CV were also analyzed by FTIR (Figure 4). Several previous investigations showed that aromatic dispersants can be deposited by EPD to form particles of different shapes at the substrate surface [25,35,36]. Therefore, FTIR studies were used to confirm the deposition of PTFE. The FTIR data of as-received PVDF showed strong absorptions at 1399, 1179, and 873 cm^−1^. The absorption at 1399 cm^−1^ resulted from wagging of CH_2_ and antisymmetric stretching of C–C bonds [37].

The bands at 1179 and 873 cm^−1^ were associated with the stretching and rocking of the CF_2_ bonds [37,38]. Those absorptions were also achieved for deposited PVDF films using CA or CV, which confirmed successful deposition of PVDF.

**Figure 5:**
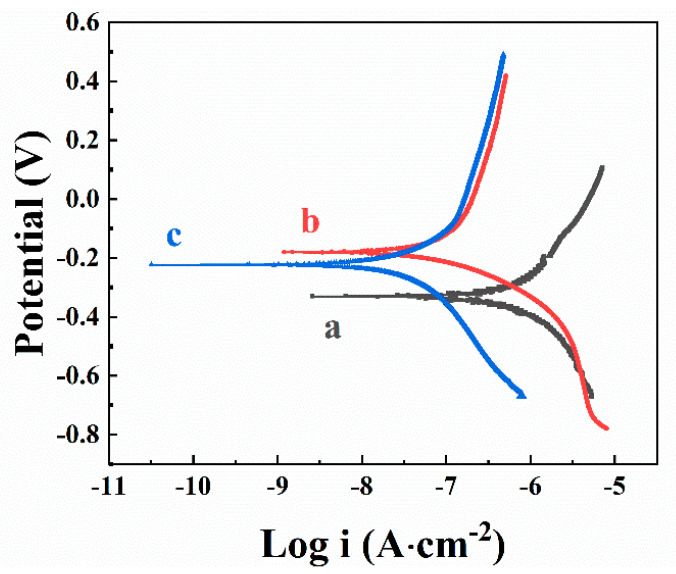
Tafel plots for stainless steel (a) uncoated and (b,c) coated from 0.5 g L^−1^ (b) CA or (c) CV solutions, containing 10 g L PVDF at a deposition voltage of 50 V during 5 min and annealed at 200 °C.

**Figure 6:**
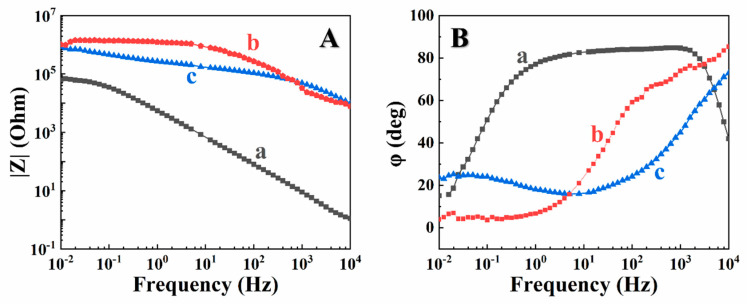
(**A**) Absolute impedance and (**B**) Phase versus frequency impedance data for stainless steel (a) uncoated and (b,c) coated from 0.5 g L^−1^ (b) CA or (c) CV solutions, containing 10 g L^−1^ PVDF at a deposition voltage of 50 V during 5 min and annealed at 200 °C.

The heat-treated PVDF films were studied for corrosion protection of stainless steel in 3% NaCl solutions by potentiodynamic analysis and electrochemical impedance spectroscopy. It was found that the annealed films offered corrosion protection of stainless steel (304-type) substrates. Testing results were presented in Tafel plots (Figure 5). Compared to the bare substrates, the coated samples showed significantly higher corrosion potential and lower anodic current (Figure 5). The calculated corrosion currents of coated samples were 0.17 μA cm^−2^ and 0.083 μA cm^−2^, for films prepared using CA and CV, respectively at a deposition voltage of 50 V and deposition duration of 5 min. The lower corrosion currents for the films, prepared using CV can result from higher film mass (Figure 2). The corrosion currents decreased significantly compared to that of uncoated samples (2.6 μA cm^−2^). The PVDF-coated sample prepared using CV showed an even lower corrosion current than that prepared using CA. Bode plots in Figure 6 showed enhanced absolute impedance in a wide frequency range for coated samples. This indicated that annealed PVDF films acted as barriers for the corrosive electrolyte diffusion to the substrate, insulating the substrates from corrosion damage.

**Figure 7:**
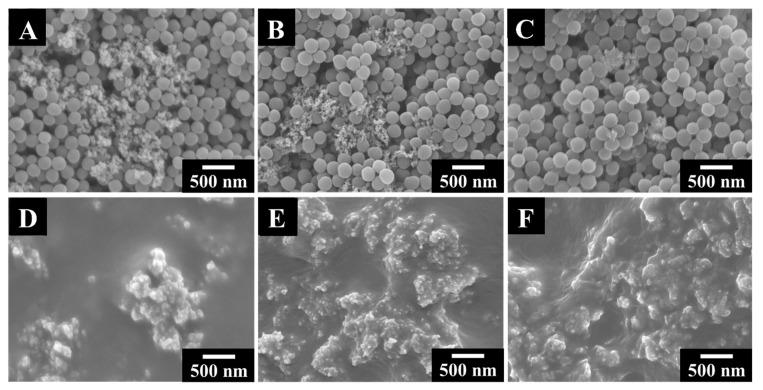
SEM images of films, prepared from 5 g L^−1^ PVDF solutions, containing (**A**,**D**) 1 g L^−1^ CA and 1 g L^−1^ MnO_2_, (**B**,**E**) 1 g L^−1^ CV and 1 g L^−1^ TiO_2_, (**C**,**F**) 1 g L^−1^ CV and 1 g L^−1^ NiFe_2_O_4_ at a deposition voltage of 50 V and (**D**–**F**) annealed at 200 °C.

The feasibility of EPD of PVDF using CA and CV as surfactants paved the way for the fabrication of nanocomposites, containing functional metal oxide nanoparticles. Following the objective of this investigation, we used MnO_2_, TiO_2,_ and NiFe_2_O_4_ nanoparticles as model metal oxide materials with advanced functionality for the fabrication of composite films. The use of CA and CV for EPD was based on different structural features of the dispersants, which facilitated their adsorption on PVDF or metal oxides. As pointed out above, the aromatic rings of the dispersant molecules facilitated their adsorption on PVDF. However, catecholate type bonding [34] involving phenolic OH groups is beneficial for adsorption on metal oxide nanoparticles. Co-deposition experiments were focused on the use of CA or CV as co-dispersants. The SEM images of the as-deposited films showed co-deposited PVDF and metal oxide nanoparticles (Figure 7A–C). Annealing of as-deposited films caused the melting of PVDF particles and the formation of crack-free continuous PVDF matrix layers with imbedded nanoparticles (Figure 7D–F).

**Figure 8:**
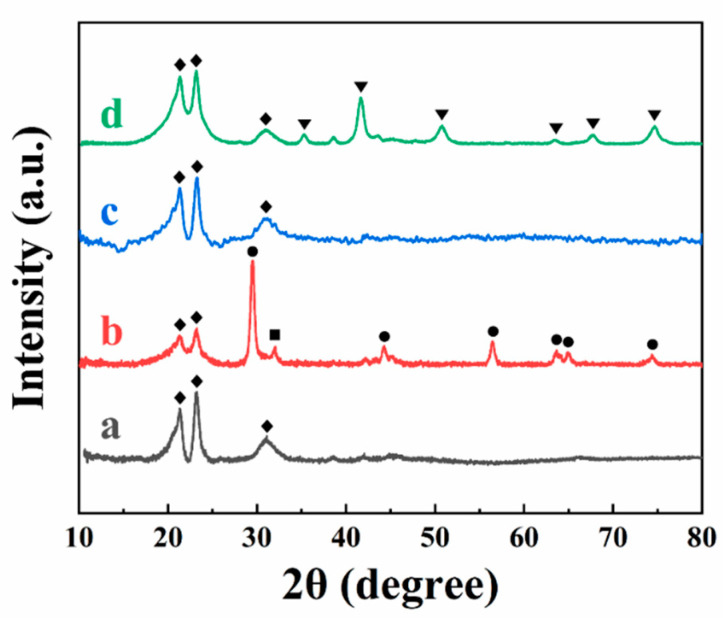
X-ray diffraction pattern of films (a) pure PVDF, (b) PVDF and TiO_2_, (c) PVDF and MnO_2_, and (d) PVDF and NiFe_2_O_4_ (peaks corresponding to ♦ JCPDS file 00-061-1403, ● JCPDS file 00-064-0863, ■ JCPDS file 04-003-0648, ▼ JCPDS file 00-054-0964). EPD conditions are similar to Figure 7.

The SEM images of the annealed films showed agglomeration of the oxide particles (Figure 7D–F and Supplementary Materials, Figure S2A–C). The typical size of the agglomerates was in the range of 200–600 nm. Such agglomeration can result from different factors, such as discharge of the particles at the electrode surface and their reduced electrostatic repulsion, hydrophobic interactions between polymer macromolecules, hydrophilic interactions of the inorganic particles, and magnetic interactions of ferrimagnetic NiFe_2_O_4_ particles. The composite films, containing PVDF and metal oxide nanoparticles were studied by X-ray diffraction method (Figure 8).

The X-ray diffraction peaks of PVDF (corresponding to JCPDS file 00-061-1403) were observed in X-ray diffraction patterns of pure PVDF (Figure 8a) and composite films (Figure 8b–d). The composite films prepared from suspensions containing TiO_2_ showed XRD peaks of the anatase phase of TiO_2_ (JCPDS file 00-064-0863) and very small peaks of the rutile TiO_2_ phase (04-003-0648). The synthesis method [32] used in this investigation allowed for the formation of amorphous MnO_2_. As a result, the X-ray diffraction pattern of the composite PVDF–MnO_2_ films showed peaks of PVDF and a broad halo related to the amorphous MnO_2_ phase. X-ray diffraction studies of PVDF–NiFe_2_O_4_ films showed peaks of NiFe_2_O_4_ corresponding to JCPDS file 00-054-0964 in addition to peaks of PVDF.

**Figure 9:**
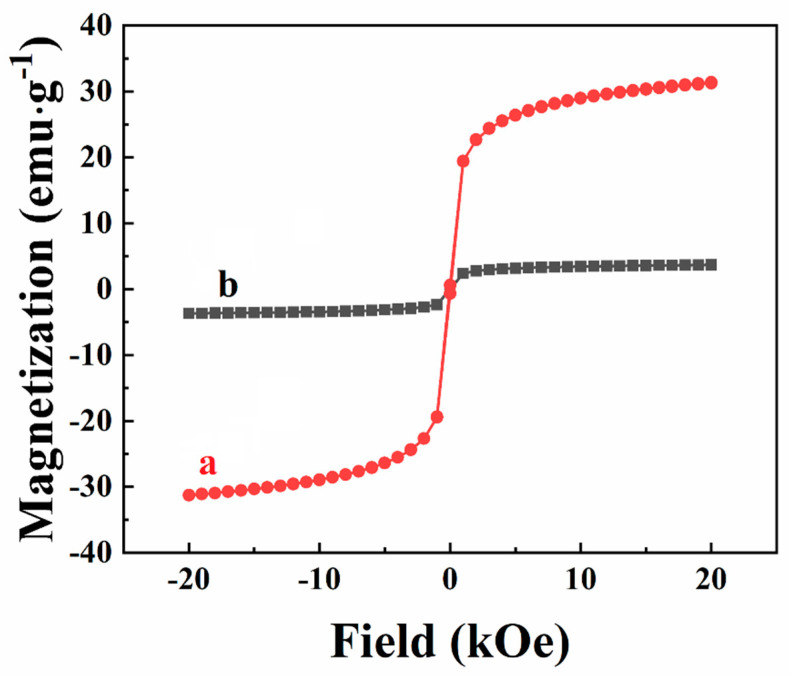
Magnetization versus magnetic field for (a) as received NiFe_2_O_4_ nanoparticles and (b) PVDF–NiFe_2_O_4_ composites prepared from 5 g L^−1^ PVDF solutions containing 1 g L^−1^ CV and 1 g L^−1^ NiFe_2_O_4_ at a deposition voltage of 50 V.

The results of this investigation showed the feasibility of co-deposition of PVDF with nanoparticles of TiO_2_, MnO_2,_ and NiFe_2_O_4_. As pointed out in Introduction, the investigation of nanocomposites containing such metal oxide nanoparticles is important for various applications. As a step in this direction, we investigated the magnetic properties of PVDF–NiFe_2_O_4_ nanocomposites. Figure 9a shows that as-received NiFe_2_O_4_ particles exhibit a superparamagnetic behavior, as indicated by the S-shaped dependence of magnetization on magnetic field. The composite materials showed a similar dependence with reduced mass normalized magnetization (Figure 9b). Therefore, the EPD method allows fabrication of nanocomposites, combining the magnetic properties of NiFe_2_O_4_ with the inherent functional properties of PVDF, such as piezoelectric, ferroelectric, corrosion protection, and other properties.

## 4. Conclusions

An EPD method has been developed for the deposition of PVDF and composite films containing nanoparticles of TiO_2_, MnO_2_, NiFe_2_O_4_, which were used as model materials with advanced functionality for the deposition of the organic–inorganic nanocomposites by the EPD method. It was found that CA and CV adsorbed on chemically inert, electrically neutral PVDF, providing dispersion and charging of the PVDF particles. The dispersant adsorption was a key factor for the successful EPD of the PVDF films. The deposition yield measurements showed that the amount of the deposited material can be controlled by the PVDF concentration in the suspensions, the deposition voltage, and the concentration of selected dispersant. The results of the potentiodynamic and impedance spectroscopy studies showed that PVDF films provided corrosion protection of stainless-steel substrates. The adsorption of CA and CV on the PVDF particles involved hydrophobic interactions, whereas the catecholate-type bonding facilitated their adsorption on the metal oxide nanoparticles. The unique ability of CA and CV to adsorb on different materials was a key factor for their use as co-dispersants for the EPD of nanocomposites. The co-deposition of PVDF with metal oxides was confirmed by the results of SEM, FTIR, and XRD studies. The ability to combine the functional properties of PVDF with the functional properties of metal oxides was demonstrated by the investigation of PVDF–NiFe_2_O_4_ films, which showed superparamagnetic properties. The approach developed in this investigation paves the way for the EPD of other chemically inert, electrically neutral polymers and their co-deposition with inorganic materials for the fabrication of novel nanocomposites with advanced functionality.

## Data Availability

The data presented in this study are available in: Versatile strategy for electrophoretic deposition of polyvinylidene fluoride-metal oxide nanocomposites.

## Supplementary Materials

The following are available online at https://www.mdpi.com/article/10.3390/ma14247902/s1. Figure S1: TEM images of as received PVDF particles and Figure S2: SEM images of annealed composite films.
