# Supplementary material for: Versatile Strategy for Electrophoretic Deposition of Polyvinylidene Fluoride-Metal Oxide Nanocomposites
Source: Materials (Basel). 2021 Dec 20;14(24):7902. doi: 10.3390/ma14247902 (PMC8707764; doi:10.3390/ma14247902)
Supplement: Supplementary file 1 [file materials-14-07902-s001.zip › materials-1474525-supplementary.pdf]

materials

MDPI

Supplementary materials

Versatile Strategy for Electrophoretic Deposition of Polyvinylidene Fluoride-Metal Oxide Nanocomposites

Qinfu Zhao [1], Xinqian Liu [1], Stephen Veldhuis [2] and Igor Zhitomirsky [1,*]

[1] Department of Materials Science and Engineering, McMaster University, Hamilton, ON L8S 4L7, Canada; zhaoq36@mcmaster.ca (Q.Z.); liux234@mcmaster.ca (X.L.); zhitom@mcmaster.ca (I.Z.)
[2] Department of Mechanical Engineering, McMaster University, Hamilton, ON L8S 4L7, Canada; veldhu@mcmaster.ca
*Correspondence: zhitom@mcmaster.ca

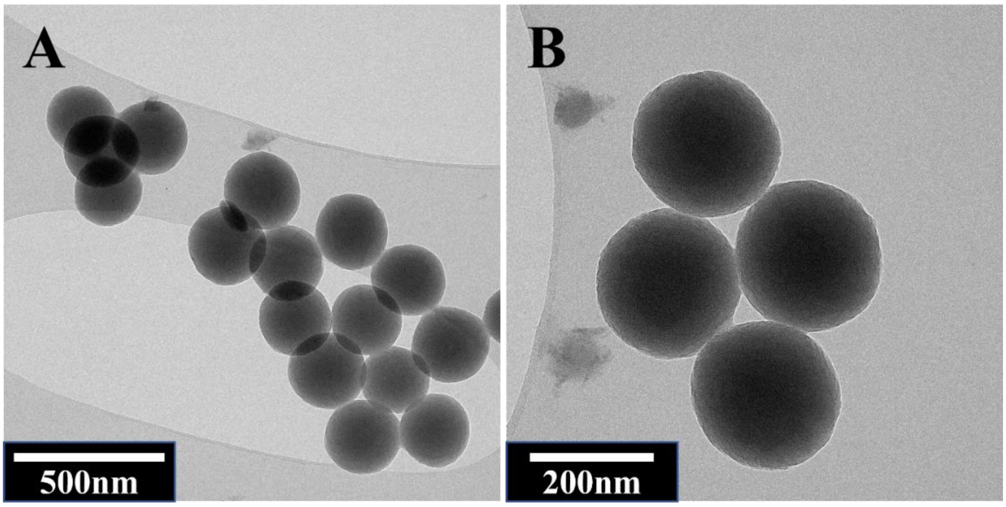

Figure S1. TEM images at different magnifications of as-received PVDF particles.

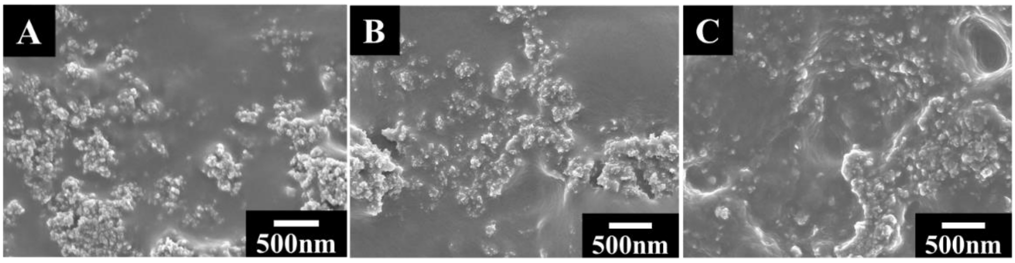

Figure S2. SEM images of films, prepared from 5 g L^{-1} PVDF solutions, containing (A) 1 g L^{-1} CA and 1 g L^{-1} MnO_2, (B) 1 g L^{-1} CV and 1 g L^{-1} TiO_2, (C) 1 g L^{-1} CV and 1 g L^{-1} $NiFe_2O_4$ at a deposition voltage of 50 V annealed at 200°C.

Citation: Zhao, Q.; Liu, X.; Veldhuis, S.; Zhitomirsky, I. Versatile Strategy for Electrophoretic Deposition of Polyvinylidene Fluoride-Metal Oxide Nanocomposites. *Materials* **2021**, *14*, 7902. https://doi.org/10.3390/ma14247902

Academic Editor: Andrei V. Petukhov

Publisher's Note: MDPI stays neutral with regard to jurisdictional claims in published maps and institutional affiliations.

Copyright: © 2021 by the authors. Licensee MDPI, Basel, Switzerland. This article is an open access article distributed under the terms and conditions of the Creative Commons Attribution (CC BY) license (https://creativecommons.org/licenses/by/4.0/).
